# Understanding Healthy Eating Habits of Elderly People in a Geriatric Center in Kirkuk City: A Cross-Sectional Study

**DOI:** 10.7759/cureus.58801

**Published:** 2024-04-23

**Authors:** Hanadi Ghalib, Nazar Mahmood

**Affiliations:** 1 Department of Community Health Nursing, College of Nursing, University of Kirkuk, Kirkuk City, IRQ

**Keywords:** kirkuk city, geriatric center, healthy eating, elderly, understanding

## Abstract

Background

A person's nutritional knowledge has a great influence on their eating habits and nutritional status. Identifying knowledge gaps can lead to educational efforts to reduce the probability of malnutrition and encourage healthy aging. This study aimed to assess older people's knowledge of healthy eating and identify associated factors in the Geriatric Center in Kirkuk City.

Methods

This was a cross-sectional study conducted from November 5, 2023, to February 25, 2024, at the Geriatric Center in Kirkuk City on 25 older adults who were targeted at the only specialist center in Kirkuk City. All center residents were included (≥60 years old and without severe cognitive impairment). A non-probability, purposive sample was employed in the study. Data were collected using an interview-structured questionnaire. The structured questionnaire consisted of two parts. The first part focused on socio-demographic variables such as age, gender, marital status, education level, and type of work before admission to the center, as well as smoking status, walking, and medical history. The second part related to assessing older people's knowledge of healthy eating using 17 items. Data entry and analysis were performed using SPSS (v 26; IBM Corp., Armonk, NY, US). Data are reported as frequency and percentage and the chi-square test/Fisher exact test was used for categorical variables. The significance level for statistical analysis was set at p<0.05.

Results

Of the 25 participants included in the study, 20 (80%) were men and 5 (20%) were women. Approximately two-thirds of the 17 (68%) were between 60 and 69 years old, with a mean age of 69.44 ± 5.71 years. Of the 25 older people surveyed, 16 (64%) had low knowledge of healthy eating while 9 (36%) had high knowledge. Knowledge level was significantly associated with age (p = 0.001), gender (p = 0.040), education level (p = 0.006), and smoking status (p = 0.037).

Conclusions

In the geriatric center in Kirkuk City, the level of knowledge of healthy eating was low. The knowledge level of people tends to be related to factors like age, gender, education level, and smoking status. This, in turn, requires implementing educational programs by healthcare providers at the center to promote knowledge and understanding of healthy diets and practices.

## Introduction

A balanced diet ensures that the body receives the necessary macronutrients to meet its energy needs without overeating and provides vital water and micronutrients for optimal health [1]. Maintaining good health into old age requires diets low in sugar, salt, alcohol, and solid or saturated fats, as well as plenty of fruit, vegetables, whole grains, legumes, nuts, and lean meat [2]. Age-related cognitive decline, cardiovascular disease risk, bone and physical health, and meaningful life experiences have improved among older adults who follow healthy eating habits [3]. However, poor nutrition in older adults is linked with negative functional and medical consequences, including increased mortality, longer hospital admissions, physical weakness, and a higher requirement for intensive care [4].

Understanding ideas and practices related to health and nutrition, for example, nutrition and disease, nutrition and health, and nutritional standards and recommendations, is commonly referred to as nutritional knowledge [5]. To prevent malnutrition and maintain health, older people need a comprehensive understanding of nutrition [6]. There is evidence to confirm that eating habits and nutritional knowledge are closely related; a recent study provided this confirmation of this relationship, showing the significant influence of a person's nutritional knowledge on eating habits and nutritional status, so recognizing the knowledge gaps can likely lead to educational initiatives that reduce the likelihood of nutritional deficiencies and promote healthy aging [7]. Many factors can make it challenging for older people to eat healthily, including limited financial resources, limited mobility, lack of social support, social isolation due to loss of close relationships, psychological problems, changes in sensory function and food availability, knowledge of nutritional and cooking methods, digestive problems, oral health, and medication factors [8,9].

Assessing the elderly population's understanding of healthy eating can play a role in encouraging nutritious food choices and nutritional strategies for the elderly; in addition, there is no nationwide survey of the older population on the level of knowledge of older people about healthy eating. This study is a starting point for a more thorough examination of older adults' nutritional knowledge. Therefore, assessing the knowledge level of older people in Iraqi geriatric centers, particularly in the Geriatric Center in Kirkuk City, and identifying associated factors is likely to fill this gap in the literature.

## Materials and methods

Design of the study

The present study employed a cross-sectional design.

Setting & duration of the study

The Geriatric Center in the city of Kirkuk, Iraq, was the setting for conducting the study. The Kirkuk Directorate of Social Special Needs reports that there was only one center for the elderly, with a total of 25 residents. The geriatric home has 15 rooms, of which 8 are occupied by elderly residents. Every room has a private bathroom and can house up to three of the elderly. Large gardens, dining halls, celebration halls, libraries, and administrative departments are all features of the senior living home. There are additional departments like a clinic, a doctor's office, and a nurses' room, dedicated to providing medical and therapeutic services. Seniors can get a free medical examination at home. Three meals are offered by the center per day, aside from snacks. The study was carried out between November 5, 2023, and February 25, 2024.

Tool of the study

The researchers developed a structured questionnaire as a data collection tool. The data were collected using an interviewing approach. The questionnaire consisted of two parts (Appendices). The first part focused on socio-demographic variables such as age, gender, marital status, education level, and type of work before admission to the center, as well as smoking status, walking, and medical history. The second part related to assessing older people's knowledge about healthy eating using 17 items. To measure older people's understanding of healthy foods, they were asked to select the “healthy,” “somewhat healthy,” “uncertain,” or “unhealthy” scale that was appropriate for each food type. A score of (1) was assigned for correct answers, and a score of (0) was assigned for incorrect and uncertain answers [10]. Scores of knowledge ranged between 0 and 17. Higher scores demonstrate a greater understanding of a nutritious diet. Because the values, as determined by a Shapiro-Wilk test, were not normally distributed, a median split method was used to transform a continuous variable into a categorical variable. With a median cut-off of 12, the knowledge level was described as high if scores were >12 and low if it was ≤12 [11].

A pilot study was carried out to assess the questionnaire's reliability before the study's implementation on five elderly participants, selected randomly. It was understood that these participants would be part of the final sample due to the small sample number. The test-retest method was used to determine reliability. The result was 0.91. A panel of over 10 experts evaluated the questionnaire's content validity. After obtaining official approval from the Kirkuk Directorate of Social Special Needs, the elderly confirmed their willingness to participate in the research by completing the questionnaire.

Sampling & sample

The total population at Kirkuk Geriatric Center consisted of only 25 elderly people, of whom 20 were men and 5 were women. All residents of the center were included (≥60 years old and without severe cognitive impairment) and were selected using the non-probability sampling method (a purposive sample). All the older people in the center agreed to participate in the study and were only included once it was ensured that they met the eligibility criteria.

Ethical consideration

The Ethics Committee of the College of Nursing, University of Kirkuk, approved the study. The administration of the Geriatric Center in Kirkuk City received official approval (No.16350/ 2023,11,19) from the Kirkuk Directorate of Social Special Needs to conduct the study. Verbal consent was obtained from each participant before data collection.

Statistical analysis

A statistical package for social science (SPSS 26) was used to analyze the data. Both a descriptive approach (frequency and percentage) and an inferential approach (chi-square/Fisher's exact test) were used. Chi-square/Fisher's exact test is used to look for relationships between variables. Fisher's exact test is only appropriate for small sample sizes and is used to compute connections using contingency tables. The significance level for statistical analysis was set at p<0.05.

## Results

Of the 25 participants involved in the survey, 20 (80%) were men and 5 (20%) were women. Approximately two-thirds of them (17; 68%) were in the 60-69 age range, with a mean age of 69.44±5.71 years. Twenty (80%) were widowed, and 19 (76%) were illiterate (Table 1).

**Table 1:** Distribution of certain sociodemographic profiles for the study sample (n = 25) Data were presented as number (n) and percentage (%) for discrete variables and as mean ± standard deviation (SD) for continuous variables.

Variables	n	(%)
Age (Years)		
60-69	17	(68.0)
70-79	5	(20.0)
≥80	3	(12.0)
Mean (SD) 69.44 ± 5.71		
Gender		
Male	20	(80.0)
Female	5	(20.0)
Marital status		
Single	3	(12.0)
Divorced	2	(8.0)
Widowed	20	(80.0)
Educational level		
Illiterate	19	(76.0)
Read and write	1	(4.0)
Primary school	1	(4.0)
Intermediate school	1	(4.0)
Secondary school	3	(12.0)

Of the 25 older people surveyed, 16 (64%) had low knowledge about healthy eating while 9 (36%) had high knowledge. The knowledge level of the elderly was significantly associated with age (p = 0.001), gender (p = 0.040), education level (p = 0.006), and smoking status (p = 0.037) (Table 2).

**Table 2:** Association of sociodemographic, lifestyle characteristics, and health status of the elderly (n = 25) and the level of knowledge *Fisher's exact test was used to test the relationship between certain sample profiles and knowledge levels. The p-value was considered significant at p<0.05. Knowledge level was described as high if >12 and low if it was ≤12.

Variables	Low knowledge	High knowledge	P value
	n= 16 (64%)	n= 9 (36%)	
Age (Years)			
60-69	15 (60%)	2 (8%)	0.001^*^
70-79	1 (4%)	4 (16%)	
≥80	0	3 (12%)	
Gender			
Male	15 (60%)	5 (20%)	0.040^*^
Female	1 (4%)	4 (16%)	
Marital status			
Single	1 (4%)	2 (8%)	0.464^*^
Divorced	1 (4%)	1 (4%)	
Widowed	14 (56%)	6 (24%)	
Educational level			
Illiterate	15 (60%)	4 (16%)	0.006^*^
Read and write	0	1 (4%)	
Primary school	1 (4%)	0	
Intermediate school	0	1 (4%)	
Secondary school	0	3 (12%)	
Type of job prior entering to the center			
Governmental	1 (4%)	1 (4%)	0.326^*^
Free or private job	7 (28%)	1 (4%)	
Jobless	6 (24%)	6 (24%)	
Retired	2 (8%)	1 (4%)	
Smoking status			
Current smoker	14 (56%)	6 (24%)	0.037^*^
Former smoker	2 (8%)	0	
Never smoker	0	3 (12%)	
Practice walking			
Never	3 (12%)	1 (4%)	1.000^*^
Sometimes	11 (44%)	7 (28%)	
Always	2 (8%)	1 (4%)	
Medical conditions			
Present	14 (56%)	7 (28%)	0.602^*^
Absent	2 (8%)	2 (8%)	

## Discussion

The study aimed to assess older people's knowledge of healthy eating and identify contributing factors at the Geriatric Center in Kirkuk City. The results showed that the elderly residents of the center had low knowledge of healthy eating. To the best of our knowledge, this is the first study that examines how much the elderly in Iraq, specifically in Kirkuk City, know about healthy eating.

Acquiring knowledge and understanding is the first essential component of developing healthy behavior [12]. The current study's results show that almost two-thirds of older people (64%) had little knowledge about healthy eating. The results obtained are slightly higher than studies conducted in other countries; For example, in Ghana, only 5.8% of older people had low knowledge [13], in Indonesia 43.3% [14], and 52.8% in Jordan [6]. Possible reasons for the observed variations in results could be attributed to the differences in education levels, differences in sample size, and lack of nutritional advice or information from healthcare providers working at the center.

Consistent with an Egyptian study, the knowledge level of older people was not associated with marital status, type of work, and health problems [3]. In contrast, knowledge of healthy eating was significantly associated with levels of physical activity and independence among older people in another study [15]. The differences could be attributed to the sample size within the center and the fact that the majority of the elderly only practiced walking as their primary form of physical activity. Moreover, age and educational level were also significantly associated with levels of knowledge. This is consistent with the results of another study conducted in five European countries, which showed that age and education level have independent and significant associations with knowledge [15]. Long-standing eating habits and a lack of knowledge of healthy eating are common among older, extremely uneducated people. A possible explanation for this could be that individuals with higher levels of education understand dietary guidelines and are more aware of the importance of food for overall health [16]. It is therefore generally accepted that education improves a person's ability to understand and retain various types of information [17].

The gender of the elderly was another factor that influenced their level of knowledge. A similar result was also achieved [6]. Another research found significant differences in nutritional knowledge index scores for gender, with women having higher knowledge scores compared to men. A possible explanation for this could be that women are more interested in nutrition-related topics because they do more household tasks such as food preparation and food selection [4]. Level of knowledge was also influenced by smoking status. The result, however, does not agree with that of another study [15] but there may be some reasons for this difference such as the setting of the study, the number of research participants, and the cultural background. Undoubtedly, a person's food quality and nutritional health are determined by their knowledge of proper nutrition, especially in high-risk groups such as the elderly. Obtaining this information is the first step to maintaining and promoting health. Therefore, good nutrition knowledge is essential [18]. 

The study design and small sample size were important limitations. First, the study is limited by the fact that the determination of cause and effect is not supported by the cross-sectional design. Second, given the relatively small sample size, the conclusions are tentative. Therefore, future studies should aim for a larger study with a larger sample size.

Among the strengths of the study, this is the first study that examines how much the elderly in Iraq, specifically in Kirkuk City, know about healthy eating. In addition, the study samples are residents in the geriatric center in Kirkuk City, considered a vulnerable group in the community, which is another strength of this research.

## Conclusions

The level of knowledge of healthy eating was relatively low among older people in the Geriatric Center in Kirkuk City. Factors such as age, gender, education level, and smoking status appear to be related to older adults' knowledge levels. This, in turn, requires offering healthy, nutritious types of foods at the center, education about the hierarchy of foods, regular checkups, control over the meals presented by health providers in the center, and the implementation of educational programs by healthcare providers at the center to promote knowledge and understanding of healthy diets and practices.

## Appendices

The questionnaire

Part I: Sociodemographic Data

1. Age in years                    …………. Years

2. Gender

- Male           □

- Female       □

3. Marital status

- Single         □

- Married      □

- Divorced    □

- widowed    □

4. Educational level

- Illiterate                           □

- Read and write               □

- Primary school               □

- Intermediate school       □

- Secondary school          □

- College and above         □

5. Type of job prior entering to the center

- Governmental               □

- Free or private job        □

- Jobless                          □

- Retired                           □

6. Smoking status

- Current Smoker             □

- Former smoker              □

- Never Smoked               □

7. Do you practice walking?

- Never                              □

- Sometimes                     □

- Always                            □

8. Medical conditions

- Present                           □

- Absence                         □

Part II: Knowledge of the Elderly Regarding Healthy Eating

**Table 3:** Knowledge of the elderly regarding healthy eating Sources: * [19]; ** [20]; *** [21]; **** [22]; ***** [23]

	Item	Healthy	Healthy somewhat	Uncertain	Unhealthy	Key answer
1.	Beans, peas, and Lentils*	□	□	□	□	Healthy
2.	White bread**	□	□	□	□	Unhealthy
3.	Fruits rich in vitamin A (e.g., apricot, watermelon, olives, and peach)*	□	□	□	□	Healthy
4.	Fruits rich with vitamin C (e.g., orange, kiwifruit, and tomatoes)*	□	□	□	□	Healthy
5.	Orange vegetables such as carrots and sweet potatoes *	□	□	□	□	Healthy
6.	Fat-free or low-fat milk and cheese*	□	□	□	□	Healthy
7.	Tea and coffee in limited amounts***	□	□	□	□	Healthy
8.	Boiled eggs*	□	□	□	□	Healthy
9.	Fatty fish (e.g., salmon, and tuna steak)*	□	□	□	□	Healthy
10.	Dark leafy greens (e.g., Spinach, kale, broccoli)*	□	□	□	□	Healthy
11.	Nuts and seeds*	□	□	□	□	Healthy
12.	Lower-fat dairy products such as milk and yogurt*	□	□	□	□	Healthy
13.	Lean meat (e.g., beef, lamb)*	□	□	□	□	healthy
14.	White meat ( e.g., chicken)*	□	□	□	□	Healthy
15.	Whole grains (e.g., oatmeal, whole-wheat bread, and brown rice)*	□	□	□	□	Healthy
16.	Low sodium soups****	□	□	□	□	Healthy
17.	Sweets*****	□	□	□	□	Un Healthy
